# Physicochemical Properties and Surface Characteristics of Ground Human Teeth

**DOI:** 10.3390/molecules27185852

**Published:** 2022-09-09

**Authors:** Katarzyna Sarna-Boś, Patrycja Boguta, Kamil Skic, Dariusz Wiącek, Paweł Maksymiuk, Jarosław Sobieszczański, Renata Chałas

**Affiliations:** 1 Department of Dental Prosthetics, Medical University of Lublin, Chodźki 6, 20-093 Lublin, Poland; 2 Institute of Agrophysics, Polish Academy of Sciences, Doświadczalna 4, 20-290 Lublin, Poland; 3 Department of Oral Medicine, Medical University of Lublin, Chodźki 6, 20-093 Lublin, Poland; 4 Preclinical Dentistry Lab, Medical University of Lublin, Chodźki 6, 20-093 Lublin, Poland

**Keywords:** ground teeth, crystallinity, FTIR spectroscopy, ICP OES method, SEM-EDX method

## Abstract

Enamel, dentin and cementum apatite has a complex composition. The lack of complete reports on the chemical composition of all tooth tissues together and the need to create a modern biomaterial that reproduces the correct ratio of individual tooth mineral components prompted the authors to undertake the research. A detailed evaluation of the micro- and macro-elements of tooth powder, using various methods of chemical analysis was conducted. All four groups of human sound teeth were crushed using the grinder. A fine powder was implemented for the FTIR (Fourier Transform Infrared Spectroscopy), ICP (Inductively Coupled Plasma Optical Emission Spectometry) and for the potentiometric titration, SEM and mercury porosimetry analyses. The obtained studies indicate that there is no significant correlation in chemical composition between the different teeth types. This proves that every removed, crushed tooth free of microorganisms can be a suitable material for alveolar augmentation. It is essential to know the chemical profiles of different elements in teeth to develop a new class of biomaterials for clinical applications.

## 1. Introduction

Human teeth are anatomical structures built up from successive layers of hard mineralized tissues: enamel, dentin and root cement. Enamel covers the entire crown of the tooth, reaching thicknesses of several millimeters. It is the highly mineralized and the hardest tissue in the human body (number 5 on the Mohs scale), with low organic content and porosity [1]. Under the enamel, there is a softer dentin (number 2 on the Mohs scale), which is the main part of the tooth. Cement covers the root, just like enamel covers the crown. Its structure resembles the bone. Variations in mineral properties in bones and teeth contribute to the mechanical performance of these tissues.

Organic tooth matter refers to the viable parts of the teeth that consist of cells, fibers, proteins, and water. It is responsible for tissue elasticity and resistance to deformation.

The inorganic matter is mainly composed of calcium and phosphate ions providing the mechanical resistance and substance of the teeth. The inorganic to organic matter ratio is different in various tissues; these variations affect the properties of these tissues [2].

Information on the mineral distribution in mineralized dental tissues is important in providing an insight into the pattern of mineralization and maturation, and in allowing a better understanding of dental development [3]. Enamel consists of an inorganic matrix (96%), organic constituents (i.e., proteins and lipids) and water (4%), which occupy the gaps among the apatite crystals [4]. Mature dentine and cementum are also highly calcified and nearly inert, although less so than enamel. Mature dentine is about 70% mineral, 20% organic matrix and 10% water by weight and cementum, respectively [5].

Overall, 80% of the teeth structure is constituted of the inorganic part, which is hydroxyapatite (HA), a form of calcium phosphate and is also known to be present in bones [6]. Other calcium phosphates and magnesium phosphates have been identified with or without association with apatite: brushite (CaHPO_4_·2H_2_O), octacalcium phosphate (Ca_8_H_2_[PO4]_6_·5H_2_O), tricalcium phosphate or whitlockite (β-TCP, β-Ca_3_[PO4]_2_), calcium pyrophosphate dehydrate (Ca_2_P_2_O_7_), amorphous calcium phosphates, struvite (MgNH_4_PO_4_·6H_2_O), newberyite (MgH_4_PO_4_·3H_2_O) and amorphous calcium magnesium pyrophosphates [7,8].

Enamel, dentin and cementum apatite (i.e., generally biological apatite) has a complex composition. Generally, it is a carbonated hydroxyapatite (HA) with a reduced calcium content and hydroxyl groups in relation to the stoichiometric HA. On the inorganic end, HA serves as the key mineral constituent, in addition to different elements that are present in minute quantities [6,9]. Biological apatite is characterized by the presence of other ions, e.g., Mg^2+^, K^+^, Na^+^, Zn^2+^, Mn^2+^, SiO_4_^4−^ and Cl^−^, partially substituted in the crystal structure (instead of calcium ions, phosphate ions or hydroxyl ions) and partially located on the surface of crystals, in the so-called hydrated surface layer [9].

Trace elements can enter human body through multiple ways, such as food, water or exposure to the environment and can get incorporated into enamel/dentin crystals. Although their presence is confirmed within teeth, not much actual literature is available to comment upon their functional significance at the level of teeth mineralization or morphology [6,10]. Examples of ion substitution that could occur in biological apatite include substitution of calcium ions with magnesium and sodium, substitution of hydroxyl sites with fluoride and chloride and substitution of both phosphate and hydroxyl sites with carbonate. With ion substitutions, a considerable variation in apatite properties could occur, for example, magnesium substitution inhibits crystal growth, carbonate substitution increases solubility, while fluoride substitution decreases the solubility [11]. The 100% substitution of the (OH^−^) with fluorine gives fluoroapatite, but this mineral structure is found only in shark enamel. Enamel apatite, like many other biological apatites, is a carbonate fluorohydroxyapatite. Calcium ions are the main components of teeth and bone in the human body. In mineralized enamel, Ca^2+^ is the most abundant ion and can be incorporated rapidly into the enamel zone from blood [12]. Tooth demineralisation, or decalcification of enamel, is a process leading to tooth decay, caused by a deficiency of minerals, in particular calcium ions. It involves a decrease in the content of inorganic substances in the enamel. Demineralisation is a process favoured by prolonged exposure to carbohydrates or acids in the oral cavity. The decomposition of monosaccharides produces organic acids that decrease the pH in the mouth. Demineralization occurs and calcium ions are removed from the enamel. Long-term exposure to acids and sugars can damage tooth enamel and cause porosities in its structure [13]. In addition, changes in the microstructure of the tooth are responsible for the proper arrangement of the enamel prisms and disorders related to mineralisation during tooth development or congenital defects such as amelogenesis imperfecta.

One of the most important, but unfortunately often overlooked, elements is phosphate. It is the main building component of teeth and bones—as much as 85% of the entire phosphorus pool is found in these tissues. First of all, phosphorus is involved in the mineralization of teeth; therefore, it determines their hardness and mechanical resistance. Its proper concentration in the body reduces the risk of caries and gum disease.

Fluoride is incorporated into the developing enamel crystallites during enamel formation and also after the enamel is completely formed; therefore, it enables proper enamel formation. Another way that fluoride works is its effect on the metabolism of bacteria by inhibiting its important enzymes. In addition, fluoride inhibits the transfer of glucose through the cell membrane, which means reducing the nutrition, multiplication and growth of bacteria, and thus hinders the growth of bacterial plaque. Overall, presence of fluoride reduces tooth susceptibility to caries [12]. The mechanism of action of fluoride for caries control works in two phases. In the first, calcium fluoride is formed on the enamel surface, and in the second, less stable hydroxyapatite crystals are converted into more stable fluoroapatite crystals. The supply of fluoride in optimum amounts even before tooth eruption prevents disturbances in enamel mineralisation and improves the relationship between apatite crystals and the protein substance, involving the formation of carbohydrate bridges. At present, it is believed that there are three main mechanisms that explain fluoride’s anti-carious effect: improved crystallisation of calcium phosphate, reduced acid solubility of enamel and an anti-enzymatic and anti-bacterial effect. Some importance is also attributed to the relationship between proper enamel maturation and the presence of fluoride [14]. Exchange of carbonate ions for fluoride therefore lowers the solubility by at least three orders of magnitude, and fluoroapatite can withstand a pH as low as 4 without dissolution. This partly explains the high benefit of fluoride supplements in toothpastes and drinking water for caries prevention and erosion reduction in teeth [12,15]. In the case of excessive supply of fluorine compounds, fluorosis may develop [12]. The severity of the disease depends on the dose, duration and age of the individual during the exposure. A very mild form of fluorosis is characterised by small, opaque, ‘paper-white’ areas scattered irregularly on the tooth. It appears that enamel spotting, showing a clinically smooth surface, actually has numerous depressions. In the aetiopathogenesis of macular enamel, imperfect bonding of its mineral part to its organic part, caused by excessive amounts of fluoride, is of great importance [14].

Magnesium is one of the most abundant trace elements in saliva and since teeth remain bathing with salivary components, magnesium is also the most abundant trace element in enamel [6]. There were many studies concerning magnesium, and it was established that magnesium was more abundant in the dentine (Chaudhri, 3–8%) than in the enamel (Chaudhri, 1–1.1%; Mann, 0.27%) [16,17]. Calcium, phosphate and fluoride ions play an important role in the equilibrium between demineralization and remineralization processes and accordingly modify the susceptibility of tooth to caries progression. During demineralization, calcium release precedes phosphate release from enamel, dentin and cementum. Therefore, using calcium rather than phosphate to suppress the demineralization process would be effective [11]. The presence of even minuscule concentrations of such elements has been reported to influence the size and organization of apatite crystals, which in turn has an impact on the hardness of the enamel. Moreover, different trace elements have been linked to various roles, such as caries protection and caries promotion [18].

Nevertheless, the importance of trace elements in modulating tooth mineralization, development and role in carries cannot be understated, though further studies are needed to look into the detailed role of the presence of these elements within hard tissues.

The incomplete reports on the chemical composition of all tooth tissues and the ratio of individual tooth mineral components prompted the authors to undertake this research. A detailed evaluation of the micro- and macro-elements, using various methods of analysis, is very important to be able to recreate what is natural.

Considering the above-mentioned arguments, the purpose of the study was to determine the structural differences of permanent human teeth with differential chemical analysis of their particles.

## 2. Results

### 2.1. Crystalinity Index and Qualitative Description of Functional Groups Using FTIR Spectroscopy

**Figure 1:**
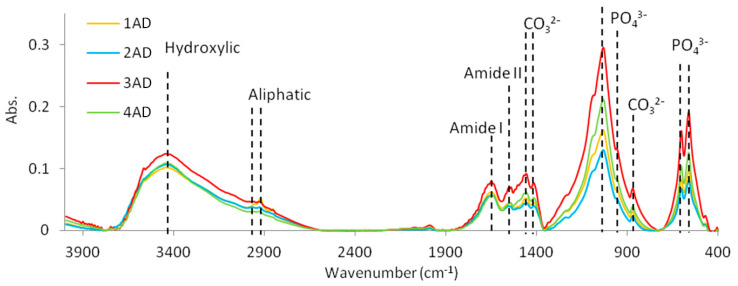
FTIR spectra of the different types of teeth (1AD-incisors, 2AD-canines, 3AD-premolars, 4AD-molars). Dashed lines indicate the main absorption bands associated with the vibration of the most important chemical groups.

The FTIR spectra were similar qualitatively in all spectral region: the main absorption bands were localized at similar wavelengths for all teeth (Table 1). Changes in spectra were found, however, for intensity of given peaks (Figure 1). The bands at ~563, 604, 960 and 1034 cm^−1^ were assigned to phosphate ions (PO_4_^3−^), while signals at ~872, 1418 and 1458 cm^−1^ were attributed to carbonates (CO_3_^2−^). Peaks located at ~1650 and 1555 cm^−1^ corresponded with the presence of amide I and amide II structures. Weak signal at ~2965 and 2928 cm^−1^ derived from aliphatic groups and broad band at ~3445 cm^−1^ from vibrations of OH or NH structures (Table 1). Bands assigned to carbonates, phosphate ions and amides decreased for 1AD (incisors) and 2AD (canines) samples. These chemical groups were the best distinguished for 3AD (premolars). Aliphatic structures were clearly observable for 1AD (incisors) sample while it disappeared for 3AD (premolars). The crystallinity of the dental hydroxyapatite showed the following trend, CI: 3AD > 4AD > 2AD > 1AD (Table 1).

### 2.2. Distribution Function of Dissociating Constants, Titrant Consumption and Acidity of Teeth Samples Obtained from Potentiometric Titration

**Figure 2:**
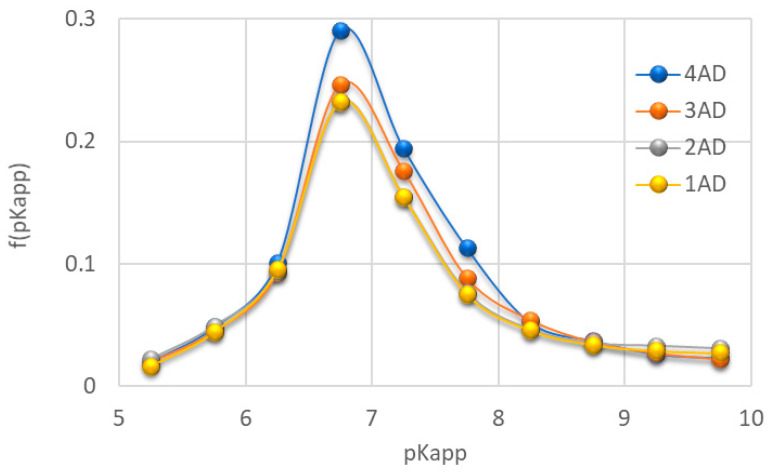
Distribution function of apparent dissociation constants for the studied teeth samples (1AD-incisors, 2AD-canines, 3AD-premolars, 4AD-molars).

**Figure 3:**
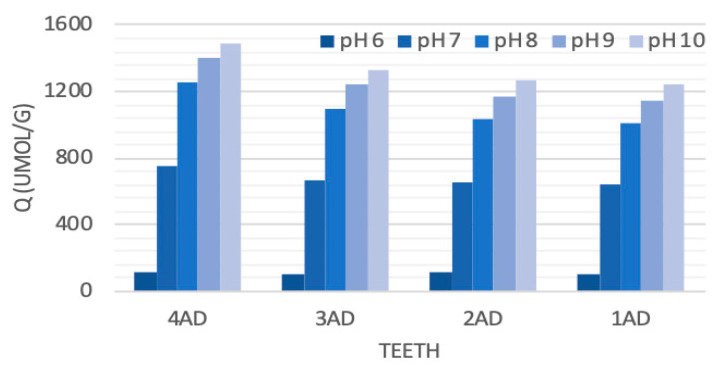
Increase in the consumption of the OH^-^ (titrant) volume during titration of different teeth at increasing pH conditions (1AD-incisors, 2AD-canines, 3AD-premolars, 4AD-molars).

Results of potentiometric titrations allowed to determine the distribution function of apparent dissociating constants (Figure 2), as well as the titrant consumption (Q) for the same pH conditions (Figure 3). It should be noted that the Q for different pHs should be treated as relative values due to the fact that titration was possible from pH 5. Obtained data show that NaOH titrant volume needed to reach pH 10 varied between the samples tested. The highest, total volume of the titrant was used for the analysis of the 4AD (molars), and the lowest one for the 1AD (incisors) and 2AD (canines), which could mean the highest and the lowest number of negatively dissociating functional groups in samples 4AD (molars), as well as 1AD (incisors) and 2AD (canines), respectively.

The highest consumption of the titrant with the slowest changes in pH was observed between pH 6.5 and 7.0. The distribution function of dissociation constants also showed a clear maximum at a pKapp of about 6.75. This indicated that the largest population of functional groups of the tested materials dissociated, generating a negative charge, in the range of pKapp 6.5–7.0. At the same time, these structures differentiated the tested material the most. The highest intensity of the dissociation constants’ distribution was observed for the 4AD (molars) sample and the lowest for the 1AD (incisors) sample. Both a significant increase in the Q, weak dynamics of pH changes and a clear maximum in pKapp~6.75 may indicate the presence of phosphate groups. These structures are strong buffers, which also explains the large volumes of titrant necessary for sample titration [19]. The buffering maximum at pH~6.75 also indicates that under these conditions the tested materials are the most resistant to pH changes caused by an acidic or alkaline environment. In this case, such resistance decreased towards: 4AD >≈ 3AD > 1AD ≈2AD.

### 2.3. Elemental Composition of the Different Types of Teeth Using ICP OES and SEM-EDX Methods

Four different chemical elements (Ca-calcium, Mg-magnesium, Na-sodium, P-phosphorus) were measured in the ICP OES tests conducted. The average levels of calcium, phosphorus and sodium were highest for 4AD (molars) samples, in the case of magnesium for 2AD (canines) samples. The Ca/P ratio indicating higher tissue mineralization was 1.89 for 1AD (incisors) and 4AD (molars) samples, while 1.86 and 1.85 for 2AD (canines), 3AD (premolars) samples, respectively (Table 2).

Table 3 summarizes the content of elements obtained in the EDX analysis for the powdered samples of various groups of teeth. All analyzed groups of powders had a very similar percentage composition of elements. The main elements were O, C, Ca, N and P. The content of Mg and Na rarely exceeded 2%, regardless of the analyzed group of teeth. The carbon, calcium and phosphorus mean values were 13.34, 23.37 and 9.80%, respectively. As in the case of the ICP OES analysis, a slightly higher Ca/P ratio was observed for the 1AD (incisors) and 4AD (molars) groups. The average ratio of these ions was 2.39.

### 2.4. Topographic and Porosity Analysis of the Teeth Powders Using SEM and Mercury Porosimetry Methods

**Figure 4:**
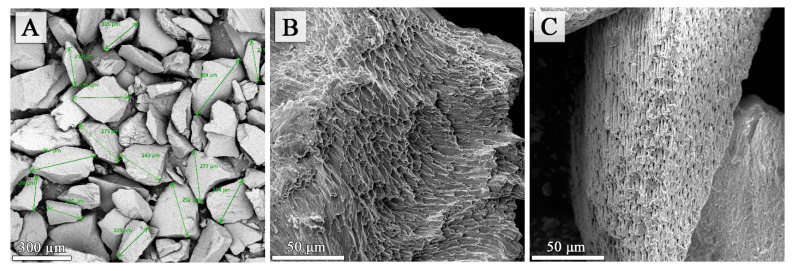
SEM images (magnification 200 and 1500×) obtained for powdered tooth samples. Images show: (**A**)—fine powder of teeth (BSE mode), (**B**,**C**) show the structures at enamel and dentin pieces surfaces (SE mode).

The SEM technique and mercury porosimetry can provide valuable information on tooth structure, especially their porosity and particle size [20]. Figure 4A shows the grains of teeth obtained by crumbling groups of teeth. In the case of fine particles of teeth, the diagonals of the analyzed grains measured at the widest point did not exceed 370 µm. In addition, SEM images allowed observing the appearance of the structures of individual teeth tissues that looked crumbled but not crushed. They contained cylindrical prisms of enamel, dentin with characteristic tubules and innervated radicular pulp (Figure 4B,C).

**Figure 5:**
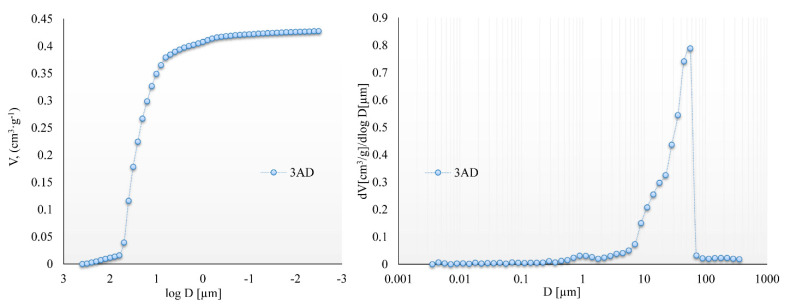
The cumulative and differential curves obtained for powdered premolars.

The characterization of the powders of ground teeth in terms of porosity was carried out by mercury porosimetry. The cumulative and differential porosimetric curves of the pore volume as a function of the equivalent pore diameter are shown in Figure 5. The differential curve revealed that the analyzed powders of ground teeth had one distinct peak related to the macropores. Peak maximum was observed for pores with a diameter of ~55 µm. In turn, the total porosity did not exceed 29%, and the pore area was 1.89 m^2^/g.

## 3. Discussion

In view of the growing interest in the use of ground teeth powder as an autogenous graft after tooth extraction, this study was conducted. Preserving sufficient bone is nowadays of paramount importance due to the widely used dental implant treatment for missing teeth. It seems essential to investigate what exactly ground teeth powder is and whether there is actually a physical–chemical justification for its use.

In this context, two characteristics—the grain size and the composition of the ground teeth powder—are of great significance.

The study by Binderman et al. demonstrates that particles of ground patient’s own teeth placed into the post-extraction alveolus become surrounded by bone matrix and undergo specific ankylosis [21]. New bone formation is stimulated by activation of mesenchymal stem cells on the rough surfaces of the biomaterial [22,23,24]. Osteoblasts create bridges between grains of different sizes and integrate with other osteoblasts to promote both proliferation and differentiation [25].

In the present study, the diagonals of the analysed grains of fine powder of ground teeth measured at the widest point did not exceed 370 µm. The greater the space between the particles, the greater the penetration of osteoblasts; the penetration of blood vessels seem to promote bone growth through osteoconduction within the pores. It has been shown that the presence of macro- and micro-pores in the graft biomaterial particles is a very important criterion [25]. They need to be greater than 100 µm to allow osteoblasts and vessels’ penetration into the pores and bone repair [26,27,28].

Bone replacement materials derived from ground teeth are considered as an attractive substitute due to their autogenous origin and favourable clinical results, which have shown that these materials provide good osteoinductive properties [25]. Based on the potential of osteoconduction, osteoinduction and osteogenesis via growth factors in the tooth and similar histogenesis between tooth and bone; a new bone graft material can be developed using inorganic and organic components of the extracted tooth [29].

Teeth and jawbones are very similar in terms of structure and chemical composition. The teeth, cartilages, nerves and bones of the maxilla and mandible all embryologically originated in the neural crest and have identical descendance [30,31,32].

Some researchers therefore propose not to dispose extracted teeth in favour of reusing them as autogenous graft material, ready for use 15 min after extraction [21,33,34].

In this study, changes in the mineral content and crystallinity of individual tooth groups were quantitatively analysed using FTIR results. It is suggested that the ratio of carbonate to phosphate reflects the level of carbonate substitution in the hydroxyapatite crystal [35]. In this study, a significant reduction in the relative carbonate content was observed in all tooth groups, indicating a change in the chemical composition of the tooth minerals.

The basic component of the tooth in terms of chemical composition is hydroxyapatite. It has been reported that natural teeth might contain fluorapatite or carbonate-apatite, which may be related to water fluoride level and the CO_2_ level in the blood and tissue fluid [36]. Carbonate-apatite is relevant to the early stages of tooth decay, as carbonate incorporation into apatite has been shown to significantly alter its properties and increase its solubility. Fluorapatite is the most stable of the dental apatites and has lower solubility and greater acid resistance than hydroxyapatite [36,37,38]. Reduced carbonate content can disrupt or disorganise the apatite lattice and regularity of atomic distribution in hydroxyapatite, thus altering its crystallinity [39].

Various cations and anions are incorporated into the cationic (Ca^2+^) and anionic (OH^−^, PO_4_^3−^) centres of the hydroxyapatite matrix. Sodium (Na^+^), potassium (K^+^) and magnesium (Mg^2+^) can substitute at the calcium position, fluoride (F^−^) and chloride (Cl^−^) at the hydroxyl position, and carbonate (CO_3_^2−^) at the hydroxyl and phosphate positions [7,40].

Both FTIR and potentiometric titrations are important sources of information showing the presence of individual functional groups of chemical compounds present in different teeth types. FTIR spectroscopy is a fast, non-destructive technique that enables rapid, sensitive and simultaneous monitoring of the different functional groups of macromolecules in biological systems [35].

This interrelationship between mineral composition, structure and properties is particularly well illustrated by the contrasts between the apatite phases in bone and tooth enamel. Bone apatite contains about twice as much carbonate as enamel apatite, and bone crystallites are only one-tenth to one-hundredth longer than enamel crystallites. Therefore, bone and enamel exhibit different order length scales. Both the smaller size of the crystallites and the higher concentration of carbonates are responsible for the much higher solubility of bone apatite than tooth enamel [41].

The main components of natural tooth tissues include light elements forming the organic polymer framework (mainly carbon (C), nitrogen (N), oxygen (O) and hydrogen (H)) and precipitates containing calcium (Ca) and phosphorus (P) with trace amounts of strontium (Sr), magnesium (Mg) and zinc (Zn) [42]. Enamel and dentin analysis by X-ray energy dispersive spectroscopy (EDS) also revealed the presence of other elements, such as Na, Cl and Mg in small amounts [5,43].

In the present study, the determination of the mineral composition of teeth by inductively coupled plasma optical emission spectrometry reveal not significant differences in the elemental composition of the individual teeth types grains. More calcium as well as phosphorus was observed in both molars and premolars. This may be related to the higher tooth weight, an increased ratio of the mineral part to the non-mineralised part. Similarly, the EDS analysis did not reveal any significant differences for grinded samples of different tooth groups. All analysed groups had a very similar percentage composition. The dominant elements were O, C, Ca, N, P. The content of other elements, such as Mg and Na, rarely exceeded 2%, regardless of the tooth group analysed.

The Ca/P ratio ranged from 2.36 for canines to 2.40 for incisors and molars in the analysed groups of crushed teeth. A higher ratio of these ions indicates a greater degree of mineralisation of the enamel. Studies using a similar method have shown that for healthy enamel, the Ca/P ratio ranges from 1.8 to 2.3, while for decayed tissue its value clearly decreases [44]. In the study by Calvo-Guirado et al., the EDX method was used to determine the elemental composition of dentin particles and a Ca/P ratio of 1.67 +/− 0.09 was obtained, which is similar to that found in synthetic hydroxyapatite HA. Traces of magnesium, which is known to be an impurity in calcium phosphate as a raw material, were also found [25].

Magnesium plays an important role in tooth formation. It is involved in the hydroxyapatite structure, which is then unmodified, but the solubility of the mineral with a higher Mg (and also Na) content increases. Unfortunately, Mg is also a component of the enzymes of the bacteria that initiate caries and then its role is only negative [16].

In the study of the chemical composition of each crushed tooth carried out by Calvo-Guirado et al. using the SEM-EDX method, it was shown that the mean values of calcium and phosphorus were highest for molars, 25.76 and 11.37%, respectively, and lowest for incisors, 22.02 and 8.3%, respectively [25]. A similar relationship was observed in the present study, 24.72 and 10.3% for molars and 22.42 and 9.35% for incisors.

The tissue formed from the combination of bone and graft material will be a suitable place for a dental implant, even though the activation of the growth factors present in the ground dentin is delayed [21].

With the development of nanotechnology in the early 1980s, there was a huge interest in hard tissue research to imitate the synthesis of such materials in vitro. The current literature contains many papers on the application of nanotechnology in bone regeneration in dentistry [45,46,47]. Nanomaterials can be synthesised from natural or synthetic materials. The various applications of nanotechnology in dentistry range from caries treatment to prosthetics and finally to the creation of dental implant materials. Nanomaterials reduce biofilm build-up, inhibit demineralisation and can be used to eradicate caryogenic bacteria. Therefore, it is essential to know the chemical profiles of different elements in teeth to develop a new class of biomaterials for biomedical applications [48].

The use of different research methods in the present study allowed for a comprehensive ultrastructural analysis of tooth tissue in different groups of teeth.

## 4. Conclusions

The obtained detailed results indicate that there is no significant correlation in chemical composition between the different teeth types. It can prove, that every extracted tooth free of microorganisms and any pathologies can be a suitable material for alveolar augmentation. It is also important to know the accurate chemical profiles of different elements in teeth, which can be helpful in developing new materials for biomedical applications.

## 5. Materials and Methods

### 5.1. Teeth Collection and Preparation for the Analyses

**Figure 6:**
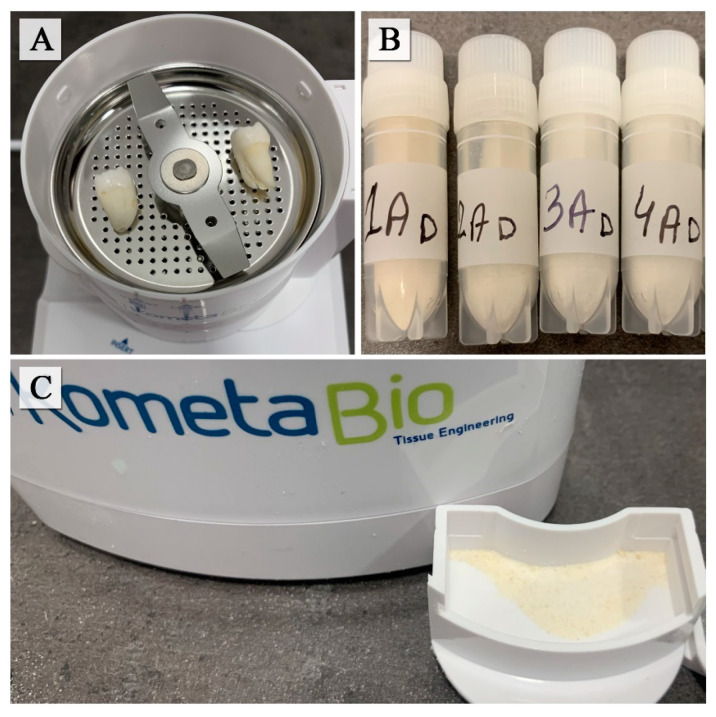
Human teeth inside the “Smart Dentin Grinder” chamber (**A**), prepared samples (**B**), compartment of particles (**C**).

**Figure 7:**
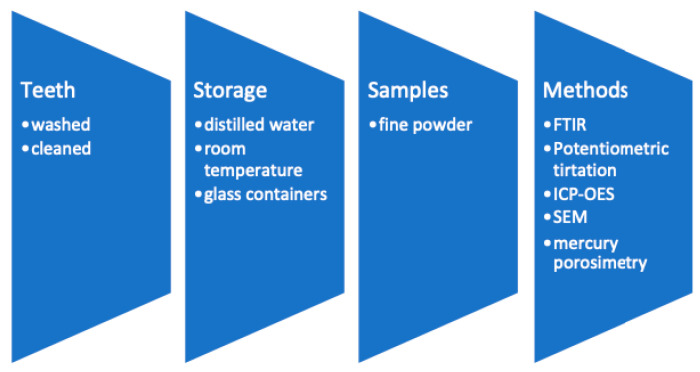
The sequence of procedures in the preparation of samples.

The study protocol was approved by the Medical University of Lublin Ethics Committee (registration number KE-0254/154/2021). The human teeth used for the examination were sound teeth without any pathologies, extracted because of trauma, periodontal disease and orthodontic reasons, A total of 50 teeth were collected from 50 donors. The teeth were washed in water to remove any traces of blood. Then they were cleaned using straight fissure carbide burs, trimming the periodontal ligament and dried with an air syringe. The teeth were divided into four groups: 1AD–incisors, 2AD–canines, 3AD–premolars, 4AD–molars. The teeth prepared in this way were stored in separate glass containers at room temperature in distilled water, which was changed daily to avoid deterioration. Teeth after being dried were crushed using the “Smart Dentin Grinder” device (KometaBio Inc., Cresskill, NJ, USA) (Figure 6A–C). A fine powder was used for the FTIR (Fourier Transform Infrared Spectroscopy), ICP (Inductively Coupled Plasma Optical Emission Spectometry) and for potentiometric titration, SEM and mercury porosimetry analyses (Figure 7).

### 5.2. Analysis of Teeth Functional Groups Using FTIR Spectroscopy

The 3 mg of each powdered sample was homogenized with 200 mg KBr of spectral purity and analyzed on a FTIR spectrometer (Tensor 27, Bruker, Billerica, MA, USA) in the form of the thin pellets. Scans were recorded in the wavenumber range of 400–4000 cm^−1^. The characteristics were obtained as an average of 256 scans at 2 cm^−1^ resolution each. Data were elaborated using smoothing function, transmittance–absorbance conversion, baseline correction and normalization. The absorption bands were described in terms of the chemical structures consisting the samples. Additionally, the crystallinity index (CI) was calculated as follows:

CI = (A_565_ + A_605_)/A_595_ (1)

where A_X_ is absorbance at given wavelength.

### 5.3. Analysis of Titrant Consumption and Distribution of Dissociating Constants of Teeth Samples Using Potentiometric Titration

The potentiometric titration was carried out using Titrino 702 SM autotitration unit, provided by Metrohm. Analyzed material was dried at 105 °C. Samples of weight equal 300 mg were placed into the 40 mL glass beakers, suspended in 25 mL of 0.1 M NaCl solution and left for 24 h. The next day, suspensions were adjusted to pH 5.0 with small volume of concentrated HCl. When the pH did not change more than 0.01 unit, the suspensions were titrated with 0.1 M NaOH prepared on the basis of 0.1 M NaCl. Titration was conducted at drift potential 10 mV/min and with maximal equilibrium time of 20 s. Reference titration curve was performed for equilibrium solution of 0.1 M NaCl by following the procedure described above. The titrant consumption (Q) and distribution function of dissociating constants f (pKapp) were calculated according to Skic et al. [49].

### 5.4. Elemental Analysis of Teeth Using Inductively Coupled Plasma Optical Emission Spectrometry

The mineral composition of teeth was determined using Inductively Coupled Plasma Optical Emission Spectrometry (ICP-OES, iCAP Series 6500, Thermo Scientific, Waltham, MA, USA). Prior to analysis of mineral elements, the mineralization of the samples (0.3 g) was conducted in a Microwave Digestion System (Berghof Speedwave, Eningen, Germany) by using optical, temperature and pressure monitoring of each sample during acid digestion in teflon vials (type DAP 100). Teeth samples were digested with 8 mL HNO_3_ (65% *v*/*v*) and 2 mL HCl (37% *v*/*v*). The mineralisation process followed a scheme: 10 min at temperature increasing from room temperature to 140 °C, 10 min at 140 °C, 15 min at temperature increasing from 145 °C to 200 °C, 10 min at 200 °C and cooling down to room temperature. The pressure did not exceed 20 bars during mineralization. After the mineralization, the clear solution, cooled to room temperature, was transferred to 50 mL graduated flasks and filled with deionized water (ELGA Pure Lab Classic) to the indicator.

The operating conditions of the ICP OES equipment were as follows: RF generator power of 1150 W, RF generator frequency of 27.12 MHz, coolant gas flow rate of 16 L·min^−1^, carrier gas flow rate of 0.65 L·min^−1^, auxiliary gas flow rate of 0.4 L·min^−1^, maximum integration time of 15 s, pump rate of 50 rpm, viewing configuration-axial, replicate-3, flush time of 20 s. Multi-element standards CCS-4 and CCS-5 (100 µg/mL in 7% HNO_3_, Inorganic Ventures, Christiansburg, VA, USA) were used for calibration of concentration of elements.

### 5.5. Scanning Electron Microscopy Combined with Energy Dispersive X-ray Spectroscopy

SEM images showing the structures and surfaces of teeth tissues were taken on a Phenom ProX scanning electron microscope (Thermo Fisher Scientific Inc., Waltham, MA, USA). Powdered teeth samples were placed on standard aluminum stubs with double-sided carbon tape. Next, samples were sputtered with a gold layer of a thickness of 5 nm to optimize the imaging (sputter coater, CCU-010 LV, Safematic GmbH, Zizers, Switzerland). The imaging was conducted in BSE or SE mode and at an accelerating voltage of 10 kV. Qualitative analysis of the chemical composition of the ground teeth was performed at an accelerating voltage of 15 kV without gold sputtering.

### 5.6. Mercury Porosimetry

Mercury porosimetry was performed on the Autopore IV 9500 apparatus (Micromeritics, Norcross, GA, USA), allowing pore characteristics, including total porosity, average pore diameter (D), total pore area (S), and pore volume (V), to be estimated. Cumulative and differential pore size distributions were obtained in the diameter range of 0.003 to 360 μm. Before analysis, powdered samples were dried at 105 °C to remove water and gaseous pollutants. The applied intrusion pressure was converted into the pore diameter by using the Washburn equation. In calculation, an assumption was made that the pore system consists of parallel, cylindrical, non-intersecting pores of different diameters, which are entirely and equally connected to the outer surface of the material and thus were available to mercury.

## Figures and Tables

**Table 1:** Frequencies of the characteristic FTIR bands of different types of teeth.

Assigned Structure	1AD	2AD	3AD	4AD
Stretching vibrations of OH, NH	-	-	3566	-
Stretching vibrations of OH, NH	3445	3445	3446	3445
Stretching vibrations of CH_3_	-	2969	-	-
Stretching vibrations of CH_2_	2924	2928	2927	2928
C=O stretching of collagen amide I	1652	1651	1651	1650
C=O stretching of collagen amide II	1553	1557	1556	1544
Stretching vibrations of CO_3_^2−^	1459	1457	1458	1458
Stretching vibrations of C-O in CO_3_^2−^	1417	1418	1418	1418
Anti-symmetric stretching vibrations of PO_4_^3−^	1034	1034	1034	1034
Symmetric stretching vibrations of PO_4_^3−^	-	-	959	960
B-type carbonate substitution (carbonate substitution for the phosphate ion) CO_3_^2−^	872	872	872	872
Stretching vibrations of O-P-O bonds in PO_4_^3-^	604	604	604	604
Stretching vibrations of O-P-O bonds in PO_4_^3^	563	563	563	563
CI (FTIR)	1.08	1.42	3.26	2.77

“-“ weak, insignificant or covered absorption band.

**Table 2:** Concentration of the macro-elements (± standard deviation) in teeth samples.

Element	Ca	Mg	Na	P	Ca/P
	(g/kg)	(g/kg)	(g/kg)	(g/kg)	
1AD	269.80 ± 3.80	5.51 ± 0.03	1.90 ± 0.01	142.60 ± 1.28	1.89
2AD	267.60 ± 3.28	6.19 ± 0.03	1.93 ± 0.01	144.10 ± 0.94	1.86
3AD	279.30 ± 5.10	5.02 ± 0.04	2.02 ± 0.02	150.90 ± 0.57	1.85
4AD	288.80 ± 2.62	4.02 ± 0.01	2.02 ± 0.01	152.60 ± 1.19	1.89
Average	276.38 ± 9.72	5.18 ± 0.91	1.97 ± 0.06	147.55 ± 4.94	1.87 ± 0.02

**Table 3:** The content of elements (mass percentage) for a fine powder made of various types of teeth.

Element	O	C	Ca	N	P	Na	Mg	Ca/P
	(%)	(%)	(%)	(%)	(%)	(%)	(%)	
1AD	45.35	13.96	22.42	7.84	9.35	0.55	0.53	2.40
2AD	45.24	13.22	23.24	7.40	9.76	0.57	0.57	2.38
3AD	45.83	13.46	23.08	6.76	9.78	0.58	0.51	2.36
4AD	45.20	12.72	24.72	6.04	10.30	0.60	0.42	2.40
Average	45.40	13.34	23.37	7.01	9.80	0.58	0.51	2.39 ± 0.02

## Sample Availability

Not available.
